# Automation of Monitoring Compliance with Technological Regulations Using the Example of the Process of Filling Petroleum Products

**DOI:** 10.3390/jimaging12080349

**Published:** 2026-08-03

**Authors:** Anatoly Sidorov, Alexey Zaripov, Ivan Tikshaev, Vladislav Pozdyshev

**Affiliations:** Department of Data Processing Automation, Tomsk State University of Control Systems and Radioelectronics, 634050 Tomsk, Russia; aleksei.v.zaripov@tusur.ru (A.Z.); ivan.d.tikshaev@tusur.ru (I.T.); vladislav.v.pozdyshev@tusur.ru (V.P.)

**Keywords:** computer vision, YOLO, regulations, control, filling of petroleum products, safety technology

## Abstract

In this paper, an approach to automating the monitoring of compliance with regulated technological operations using computer vision methods is proposed and investigated, with the loading of petroleum products considered as a case study. A distinctive feature of this work is the integration of a formalized description of the production process in BPMN notation with an object detection system, enabling not only object recognition but also interpretation of the sequence of technological actions performed by personnel. Based on the collected and annotated dataset containing more than 6000 images, a YOLOv11 neural network model was trained to monitor key stages of a technological operation. The experimental results show that the trained model provides high accuracy in detecting objects during the daytime (*mAP50* is approximately 0.98), while maintaining the ability to work in real time. The results obtained confirm their applicability in industrial conditions. The work revealed the dependence of the quality of computer vision system functioning on the illumination conditions of the production area. It has been established that at night there is a significant decrease in recognition accuracy due to the presence of glare from lighting sources directed at the camera area. The results obtained make it possible to substantiate the need to take into account lighting factors when designing video monitoring systems for technological processes. To move from the level of object detection to monitoring compliance with regulations, an algorithm for interpreting detected objects has been developed, which ensures the fixation and analysis of the sequence of operations performed. Experimental tests conducted at the existing production site have confirmed the possibility of automated detection of violations of technological regulations and an increase in the level of industrial safety. The directions for further development of the proposed approach have also been identified, including the expansion of the training sample, taking into account a variety of production scenarios, and the development of methods to increase the stability of the system in difficult light conditions.

## 1. Introduction

According to the International Labor Organization, occupational injuries and diseases are a global occurrence, with hundreds of millions of workers suffering non-fatal injuries each year, and millions dying as a result of work-related factors. Industries with a high concentration of technological operations and exposure to hazardous production factors, including mining, construction, and manufacturing, remain the most dangerous. In these conditions, digital monitoring systems are becoming particularly relevant, capable of ensuring continuous monitoring of compliance with industrial safety requirements and timely detection of deviations from regulated procedures [1].

The causes of accidents are usually deviations from established technological regulations when performing manual operations, as well as non-compliance with regulatory requirements for occupational safety and health at production facilities. Violations of regulations can lead to economic losses and dangerous or catastrophic situations.

Monitoring compliance with regulations and the sequence of actions can be carried out using various methods, one of which is visual inspection. However, it comes with a number of significant limitations. Firstly, the rate of reaction to deviations from the norm is limited by the physiological capabilities of the human visual analyzer. Secondly, observation has a high degree of subjectivity: there may be omissions of violations, underestimation of the significance of deviations, which, in turn, depends on the level of qualification of the specialist, the degree of fatigue, concentration of attention and other factors. Thirdly, this stage of the production process is resource-intensive, since it requires the involvement of qualified personnel, whose remuneration does not lead to a direct increase in the added value of products.

In this regard, digital technologies are currently becoming more widespread, in particular, computer vision systems, the use of which makes it possible to automate control and monitor tasks. They demonstrate versatility of application and can be adapted to a variety of industries and production environments.

## 2. Computer Vision Tools for Security

Computer vision devices are currently actively developing and are widely used in the identification of personal protective equipment (PPE) in industrial enterprises. In [2], a production environment was reproduced, including an extensive network of sensors, video cameras, robotic manipulators, a conveyor belt, and programmable logic controllers. The purpose of the work carried out was the automatic recognition of specific types of PPE, as well as the identification of employees with subsequent notification of the status of the presence or absence of protective equipment for each of them.

To develop the computer vision system, more than 4000 images were generated, covering a variety of production scenarios. Fast R-CNN was used as the architecture of the neural network model, which was trained with validation for 85% of the total dataset. The resulting system demonstrated an average detection accuracy of 0.74 for all classes, which is an acceptable indicator for detecting cases of lack of personal protective equipment among employees.

In [3], for the task of detecting personal protective equipment on employees, a computer vision model of the YOLO family was used and trained on a dataset containing 2500 images. The model was adapted to classify workers into four categories: those in danger, those without a protective helmet, those without a safety vest, and those who are safe.

As a result of the training, classification accuracy was achieved at 96%. When tested on a video stream, the system demonstrated its claimed effectiveness by correctly classifying personnel in real time, including providing recognition of transitions between different classes of objects.

The works [4,5,6] also describe a method for controlling PPE in production facilities. Basically, either CNN-based models or YOLO families are used for detection and classification.

The authors of [7] use short-circuit testing in the steel industry to identify occupational hazards. To create a final PPE detection system for employees, an extensive comparison of neural network models from the YOLO family was carried out, as they demonstrate the highest image processing speed. YOLO9c has demonstrated the best results in detecting security breaches.

The work in [8] is devoted to ensuring the safety of storage facilities. After analyzing the subject area, the need was identified to monitor the safety slings of containers when loading onto a truck and to prevent abnormal situations in the form of rupture or displacement of belts into a dangerous position, leading to a fall of goods during further transportation. Within the framework of the problem, the creation of a system for monitoring loading zones with automatic verification of the correctness of the installation of safety slings and monitoring the maximum weight of trucks in real time is being considered.

A two-step method is used to determine the condition of the slings. At the first stage, the YOLOv4 detection model is used to identify and locate containers in the camera’s field of view. In the second stage, the images are segmented to position and determine belt tension. The final model achieves 96.2% accuracy when detecting belts under normal operating conditions with a delay of 287 ms from the real video stream, which allows for real-time monitoring.

In the main range of security solutions in various industries, developers focus on detecting and classifying certain entities without subsequent processing. At the same time, preventive control is not provided to avoid violations before they occur. Accordingly, the task of preventing emergency situations before they occur by monitoring compliance with regulatory actions is being updated.

To automate the control of personnel actions and ensure the safety of the production process, it is necessary to analyze it, a key stage of which is the modeling of technological operations. The reproduction of the technological process in the form of a sequence of interrelated actions makes it possible to systematically identify potentially dangerous areas and areas with an increased level of risk.

Technological processes aimed at the production of products, as a rule, include a large number of operations involving the effects of harmful and/or dangerous production factors. Such processes may include mechanical, thermal, chemical and other impacts that pose a potential threat to the life and health of personnel, as well as to the environment and property.

A significant category among them is explosive and fire-hazardous production processes. In particular, the technological operation of oil filling is one of the riskiest stages of handling flammable liquids. This process is characterized by the presence of factors contributing to the accumulation of static electricity, the release of flammable vapors, possible depressurization of equipment, and errors in the actions of maintenance personnel. All of these circumstances significantly increase the likelihood of accidents, including ignition, explosion, and environmental pollution.

It is also worth noting that oil is released at various enterprises using similar algorithms of action with minor differences. The sequence of operations, as a rule, includes the arrival of a tanker truck, the implementation of preparatory measures, the immediate filling process and the subsequent departure of the vehicle. This repeatability of procedures creates prerequisites for the development of a generalized process model that can be adapted to the specifics of the regulations of a particular organization.

## 3. Technological Process Model

As part of the process analysis, a model presented in BPMN notation was developed. The filling of petroleum products was structured as a sequence of technological steps, including: (1) arrival of the tanker truck at a specialized filling site; (2) implementation of a set of preparatory measures; (3) direct filling of petroleum products; (4) departure of the tanker truck. The models were developed based on documentation that met the safety standards set by the oil company and were regulated during negotiations with the company’s employees. The final models were approved by representatives of the company, on the basis of which the test version of the system was deployed.

The organization and implementation of these operations are carried out on the basis of a clearly regulated interaction between the operator of the automated workplace and the personnel servicing the automated filling system (AFS).

**Figure 1:**
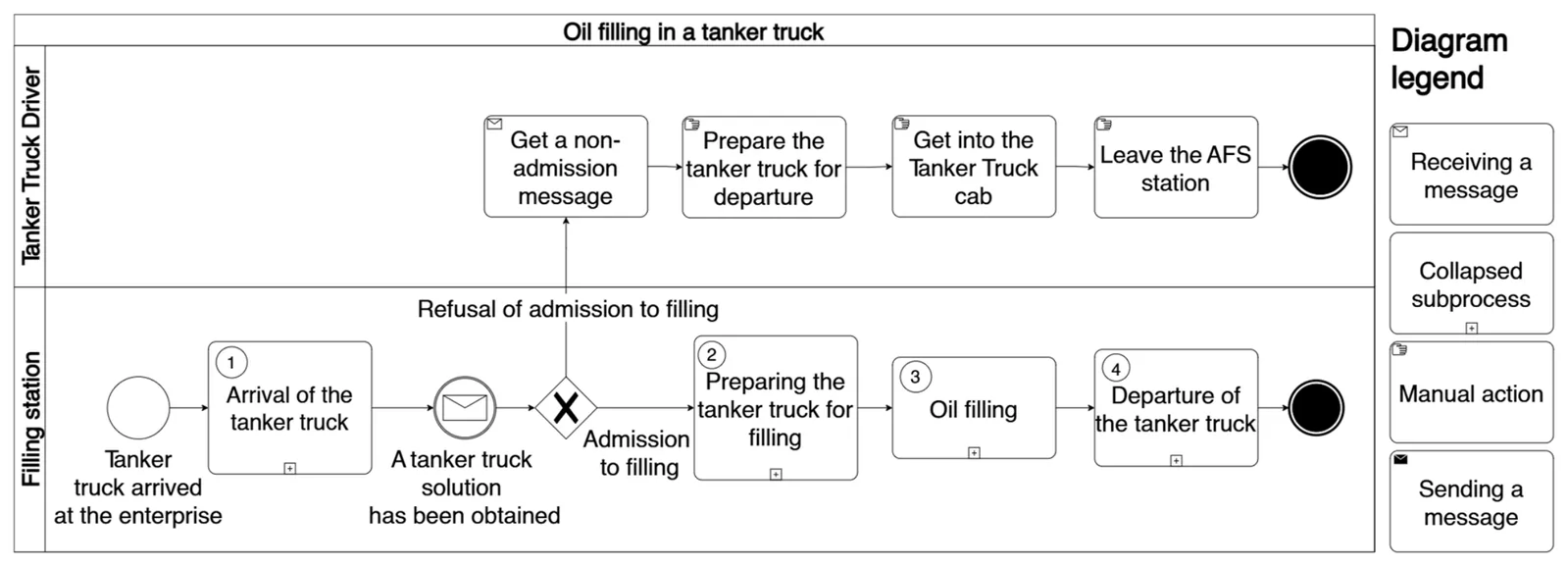
Generalized model of the oil filling business process.

Coordination of actions is carried out in strict accordance with the requirements of regulatory documentation, including industrial safety standards, technological regulations and internal instructions of the enterprise. The full cycle of oil filling into a tanker truck is defined as an activity, and the action to be monitored is defined as an event. Figure 1 shows a generalized model of a regulated technological process.

**Figure 2:**
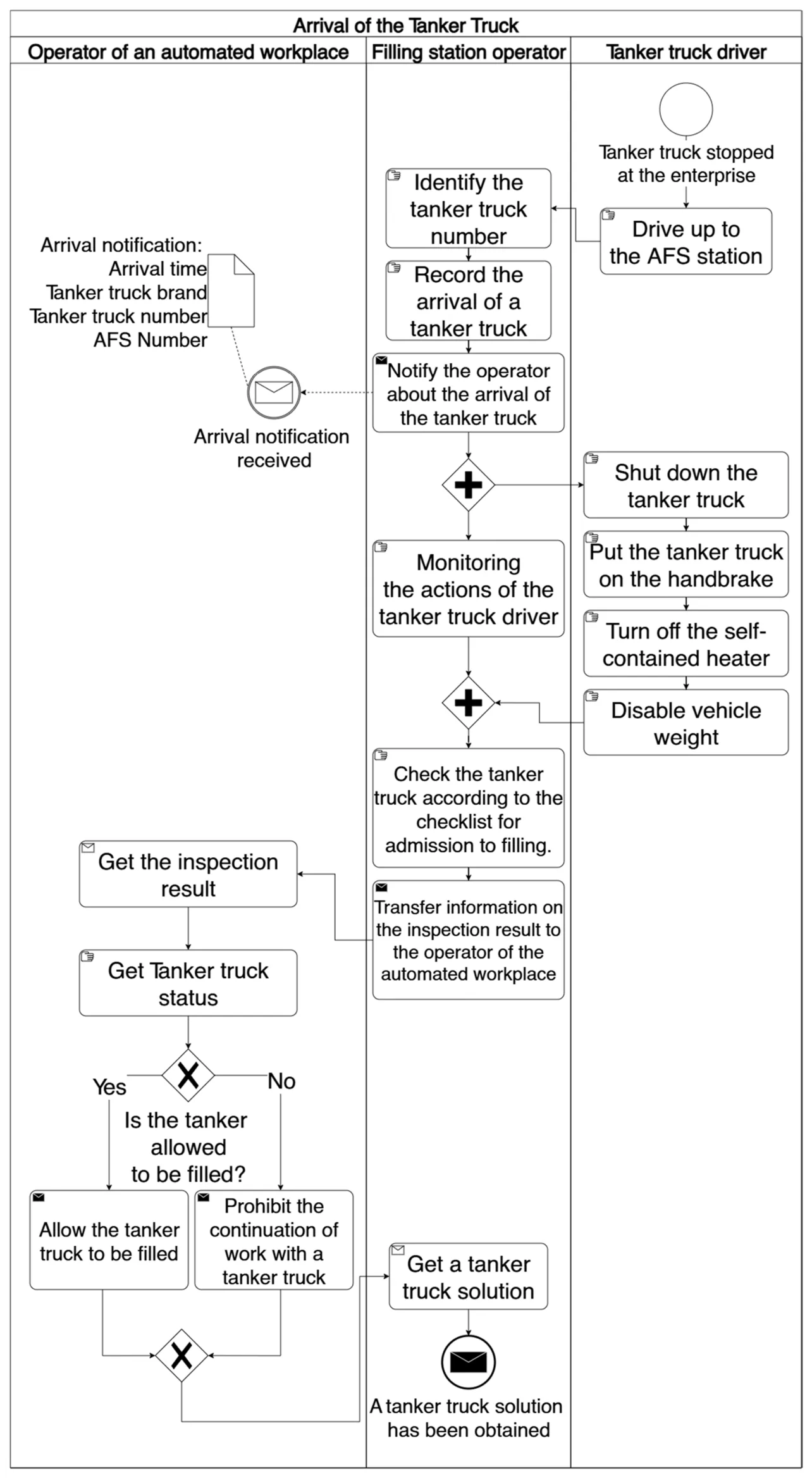
The model of the “arrival of the tanker truck” stage.

At the initial stage (Figure 2), designated as “Arrival of the tanker truck”, the vehicle enters the territory of the AFS site.

**Figure 3:**
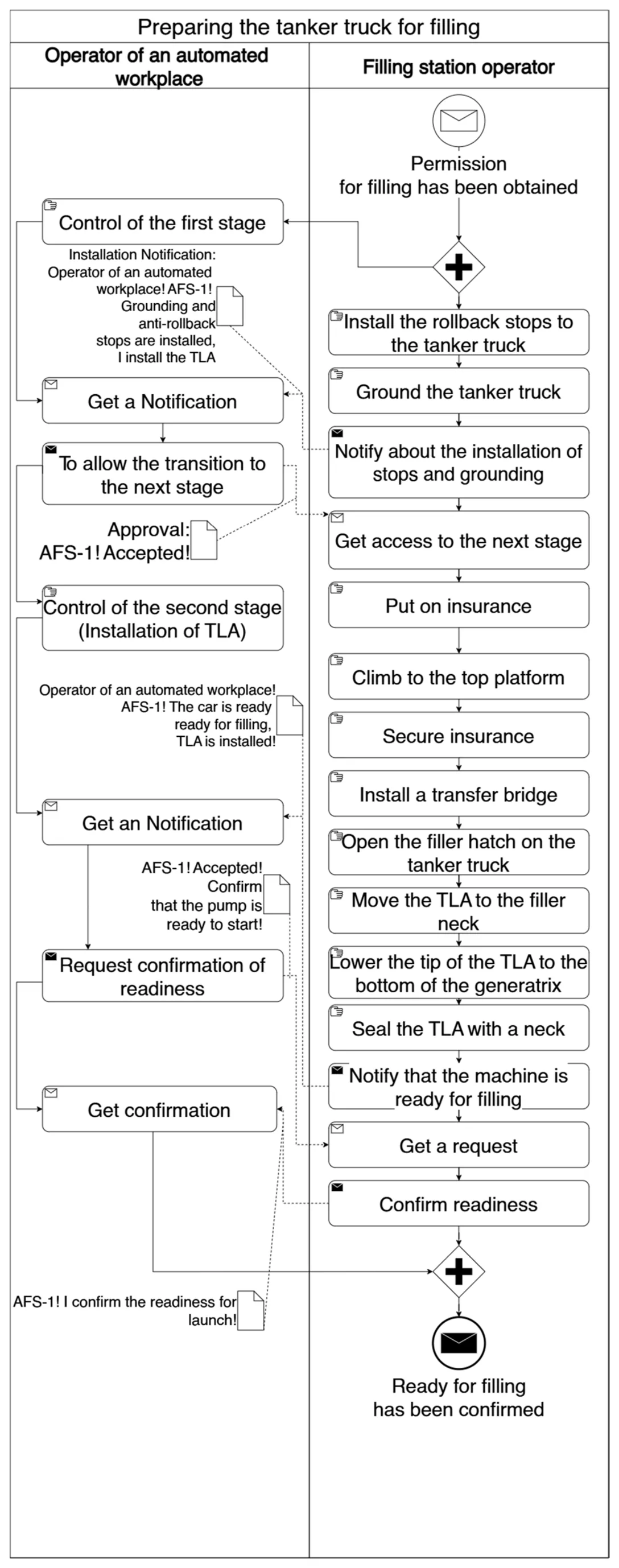
Stage model “preparing the tanker truck for filling”.

At the next stage of the technological process, the control center, located at a certain filling position, is prepared for filling operations (Figure 3).

**Figure 4:**
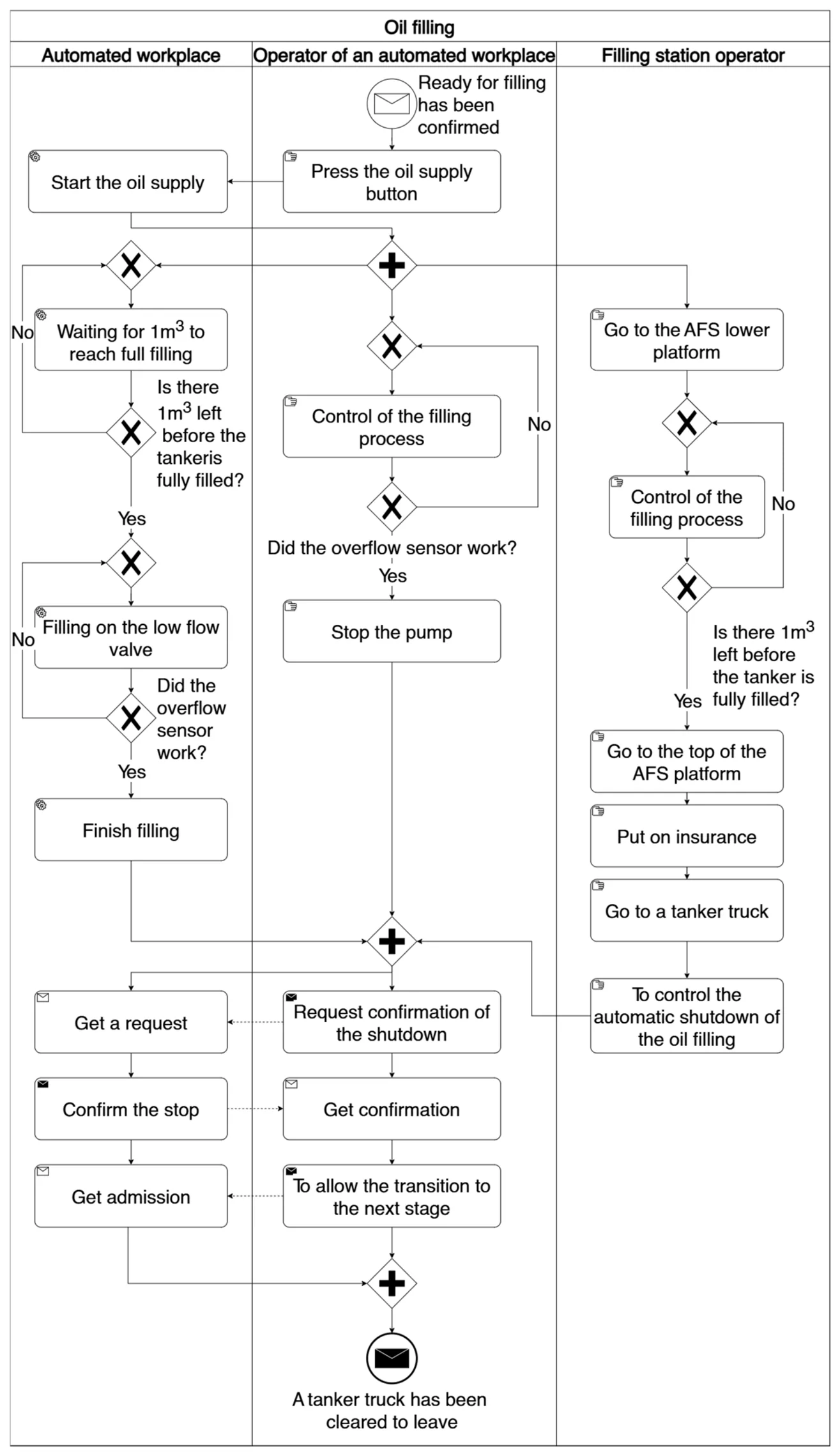
The model of the “oil filling” stage.

The next stage of the technological process involves the operation of filling oil products (Figure 4). At this stage, the operator of the automated filling system prepares the tanker truck and informs the operator of the automated workplace about the readiness of the vehicle for the start of the operation.

**Figure 5:**
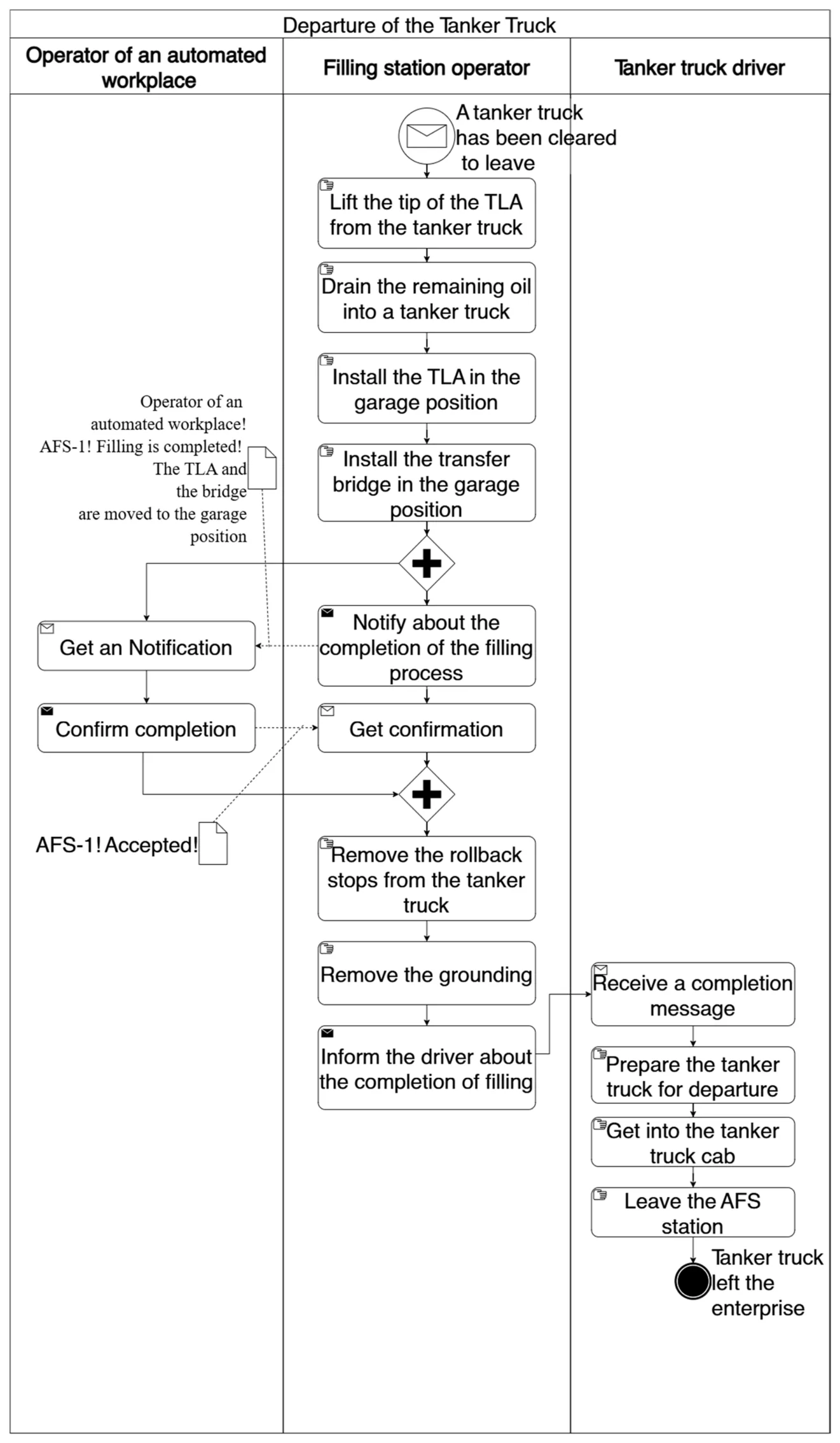
The model of the “departure of the tanker truck” stage.

At the final stage, the AFS operator performs actions to free the tanker truck (Figure 5).

It is worth noting that the actions within the process are performed simultaneously in different tracks, which indicates a high degree of parallelism of operations. This is evidenced by the presence of a large number of “And”-type gateways that structure the process, dividing the overall flow of tasks into two or more parallel directions. This approach allows the total execution time of operations to be reduced by simultaneously performing interdependent or independent tasks within their tracks.

This process is subject to automation as part of filling control by the operator of an automated workplace. Since human resources do not allow accurate tracking of manual operations, it is proposed to develop algorithms for monitoring the correctness of the sequence of actions of the AFS operator using computer vision. This will not only improve the safety of work at the production facility but also reduce the cost of paying a specialist.

To automate the process of controlling the sequence of manual operations, it is necessary to identify key events, the sequence of which the system will monitor. Table 1 summarizes the sequence of events to be monitored with the corresponding stage of the technological process.

**Table 1:** Key events to be monitored by the CV system.

The Stage of the Technological Process	Event	Controlled Parameters	Example
Arrival of the tanker truck	Tanker truck on AFS	Availability of tanker trucks on AFS	A truck with a tank is in the frame of the image (Figure 6A)
	TLA (top loading arm) in the garage position	TLA is located on the left side of the AFS	The TLA is located on the left side of the frame and forms a vertical rectangular bounding box (Figure 6A)
	The transfer bridge in garage position	The bridge is positioned vertically	The transfer bridge forms a vertical rectangular bounding box (Figure 6A)
Preparing the tanker truck for filling	The tanker truck is grounded	The presence of a ground connection signal	The AFS station sends a ground connection signal to the server
	A transfer bridge is installed on a tanker truck	The transfer bridge is in a horizontal position	The transfer bridge forms a horizontal bounding box (Figure 6B)
	TLA in the hands of the operator	The TLA intersects with the AFS operator	The bounding box of the TLA object intersects with the bounding box of the AFS Operator object; the shape of the bounding box of the TLA object changes from vertical to horizontal (Figure 6B)
	The TLA is installed in a tanker truck	The TLA is inside the tanker truck	The bounding box of the TLA object in a horizontal form (Figure 6C)
Departure of the tanker truck	TLA in the garage position	The TLA is located on the left side of the AFS	The TLA is located on the left side of the frame and forms a vertical rectangular bounding box (Figure 6D)
	The transfer bridge is in the garage position	The bridge is positioned vertically	The transfer bridge forms a vertical rectangular bounding box (Figure 6D)
	The tanker is not grounded	No ground connection signal	The AFS station does not send a ground connection signal to the server
	Tanker truck left AFS	The absence of a tanker truck on the territory of the AFS	A tanker truck is missing from the image frame (Figure 6D)

Based on the results of the process modeling, key events and relevant parameters were identified that are subject to automated control using computer vision technology.

**Figure 6:**
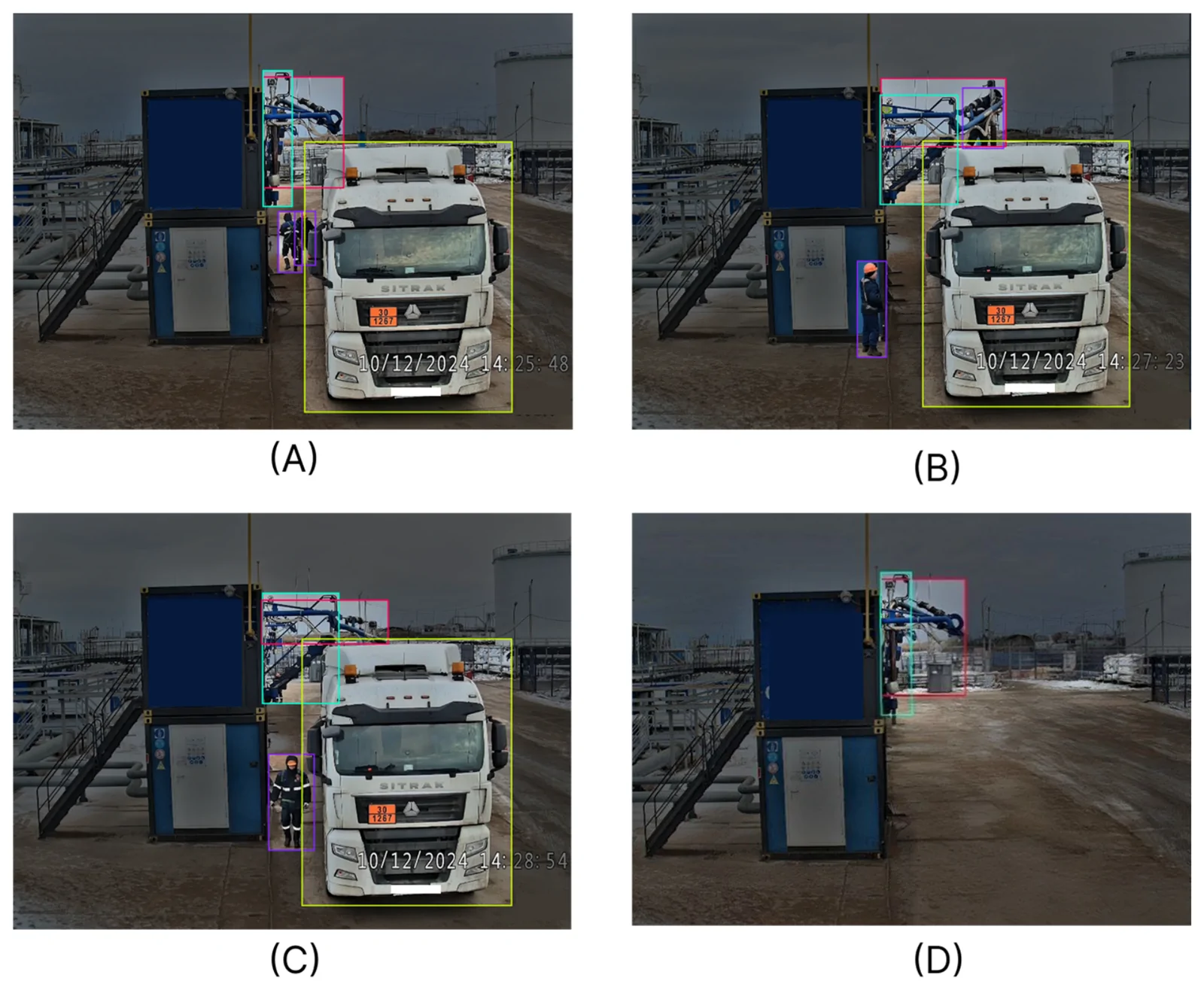
Events of the oil product filling process: (**A**) the event of the tanker truck arrival stage; (**B**) the event of the tanker truck preparation stage; (**C**) the event of installation of the overhead filling device in the tankers; (**D**) the events of the tanker truck departure stage.

The implementation of automated monitoring of these events will minimize the risk of accidents and emergencies caused by the human factor, as well as significantly increase the efficiency and safety of work at the production facility.

For the practical implementation of automated monitoring, it is necessary to develop a computer vision system based on neural networks capable of recognizing these events in real time. In this regard, the next stage of work will be the formation of a specialized dataset for training a neural network.

## 4. Formation of a Computer Vision Model

Based on the events identified during the process modeling, a dataset was formed for training a neural network using the Roboflow platform. An array of video recordings obtained directly on the production site was used as the source data. Using hand tools, key personnel were selected from this array, corresponding to the moments of recording events significant from the point of view of the system’s functioning, such as the arrival of a vehicle, the installation of a bridge, the movement of a TLA, the appearance of an operator, and other relevant actions.

The selected images were uploaded to the Roboflow software environment for the annotation procedure. During annotation, each object to be recognized by the system was assigned a corresponding category (class), which provided the possibility of subsequent training of the model on the marked-up data.

During the markup process, a problem was identified related to the annotation of the TLA, due to its small size and, as a result, low visibility in the original images. When using images in the 640 × 640-pixel format, the object (TLA) was significantly distorted due to compression artifacts and visually represented as an indistinguishable dot. To fix this problem, scaling of images to a resolution of 1920 × 1088 pixels was applied, which allowed for a clearer selection of the gander and improved the quality of the markup.

To determine the events in the image, the objects in the images were classified into the following categories: “TLA” (TLA), “person” (AFS operator), “stairs” (transfer bridge) and “truck” (truck/tanker). At the initial stage of annotation, which was carried out mainly manually, bounding boxes were manually formed on each frame of the image, covering the objects of interest. Subsequently, as the labeled data accumulated, a preliminary computer vision model was trained that could automate the annotation process.

Before starting the training, an automated orientation correction procedure was applied to the entire annotated set of images, the purpose of which was to bring all frames to a unified position of objects in the scene, regardless of the initial position of the camera. This preliminary transformation is necessary to ensure the consistency of the spatial representation of the data.

Attention was also paid to augmentation methods—the artificial expansion of the training sample through the systematic application of various image transformations. In particular, up to 10 new variants with varying distortions were generated for each training unit. This approach makes it possible to significantly increase the stability of the model to a variety of conditions typical of the real production process, where objects can appear at different angles, with unstable lighting, partial overlaps or a change in spatial location [8].

Rotations of images by 90 degrees, clockwise and counterclockwise, were used as basic transformations. This ensures that the model is invariant to the orientation of objects, especially in situations where cameras are set at different angles or the shooting point changes. For example, objects such as the “TLA” or “transfer bridge” can be visualized with any orientation, and the model must confidently recognize them regardless of their position.

Additionally, arbitrary rotations in the range from −15° to +15° were used, simulating the camera’s natural deviations, vibrations, or mounting imperfections. Due to such changes, the neural network becomes resistant to moderate disturbances in the horizontal position of the frame, which is also typical for industrial shooting conditions.

Augmentation also included mirroring images horizontally and vertically, which is especially important for production facilities where actions can occur symmetrically. Without such transformations, the model becomes sensitive to the direction of the scene, while mirror augmentations allow it to develop invariance to such symmetries.

Finally, the partial conversion of images to grayscale was aimed at reducing the dependence of the model on the color and light characteristics of the scene. Given the variable lighting conditions in production (including changing times of day, weather conditions, and lighting equipment features), discoloration of some images contributes to the development of the model’s ability to focus on structural features such as shape, contour, and proportions of objects, rather than solely on color.

The complex application of these methods significantly increases the overall stability and generalizing ability of the computer vision model, allowing it to function effectively in conditions of high scene variability and unfavorable or unpredictable shooting conditions.

After forming the critically needed set of images, a dataset with YOLO format annotation was formed. This family of neural networks was chosen because of their main advantage—the speed of operation. This architecture is designed to detect objects in images or videos in real time, which is a key indicator for monitoring potentially dangerous processes [9].

The final dataset, created to solve the computer vision problem, consists of 6820 labeled images that are intended for the learning phase. The dataset was divided according to a 70/15/15 scheme, with 4774 images used for training, 1023 images for validation, and 1023 images for testing. This distribution provides both a sufficient sample size for effective training of the model and allows for a more reliable assessment of its generalizing ability for the task of industrial safety.

The test sample includes images taken on different days, at different lighting levels, at different positions of the tanker truck and the overhead filling device, and with partial overlaps of objects. Thus, highly correlated neighboring frames were not distributed simultaneously between the training, validation, and test samples.

The YOLO v11 version was chosen for training because this version of the family is characterized by high performance with fewer parameters, which is the main criterion for production control, since the speed of the system critically affects the prevention of emergency situations [10,11,12,13].

The computer vision model was trained for 150 epochs using an early stop mechanism when the criterion of convergence of features and repeated retraining was reached. This approach made it possible to minimize the risk of retraining the neural network and preserve its generalizing ability. The size of the batch was limited by the amount of video memory used by the graphics accelerators (four NVIDIA RTX A6000 graphics cards with 48 GB of memory each). The optimal batch value of 16 provided a balance between the learning rate and the stability of the gradients, which contributed to the efficient use of computing resources while maintaining the statistical stability of the optimization process.

A comparative experiment on limited ablation was also conducted to validate the chosen configuration. Excluding geometric augmentations reduced the mean average precision (*mAP50*) from 0.857 to 0.821, while excluding augmentations related to grayscale and lighting reduced it further to 0.804 on the same dataset. On the same partition, YOLOv8n achieved *mAP50* of 0.963, YOLOv10n achieved *mAP50* of 0.974, and YOLOv11n achieved *mAP50* of 0.988, with comparable real-time performance. Additional runs showed that stable convergence can also be achieved on a single RTX A6000 GPU with a smaller batch size, albeit with increased training time. Therefore, the four-GPU configuration was mainly used to speed up the experiments, rather than being a prerequisite for system implementation.

The selection of the optimizer and its hyperparameters was carried out automatically, which made it possible to adapt the learning process to the specifics of the task being solved. Additionally, pre-trained weights were used as initialization, which helped to accelerate convergence and increase the overall effectiveness of model training.

The *mAP50* and *mAP50:95* metrics were used to assess the quality of the model, as they are key indicators widely used to analyze the effectiveness of computer vision models. It aggregates a number of parameters, including precision (*p*), recall (*r*), Intersection over Union (IoU), and average precision (*AP*), thereby providing a comprehensive description of the model’s quality.

In particular, the value 0.5 corresponds to the condition under which the prediction is considered correct if the area intersection exceeds 50%, and the value is 0.95 if this indicator exceeds 95%. *mAP50:95* aggregates the results with a sequential change in the IoU threshold from 0.5 to 0.95 in increments of 0.05, which allows an evaluation of the quality of the model not only at minimal capacity, but also under strict matching criteria.

Next, a precision(*p*)–recall(*r*) curve is calculated for each class of objects, which shows how accuracy varies depending on recall, after which the *AP* value for each class is calculated as the area under this pr curve:

(1) $$AP=\int_{0}^{1}p\left(r\right)dr$$

As a result, after obtaining the *AP* values for all classes, the final *mAP* metric is calculated, which is the average *AP* value for all classes of objects:

(2) $$mAP=\frac{1}{k}\sum_{i}^{k}AP_{i}$$

where *k* is the number of classes.

As a result, if the *mAP50* value of the model is 0.75, it means that, on average, the detection accuracy for all classes of objects (at IoU > 0.5) is 75%, which is considered a good result.

**Figure 7:**
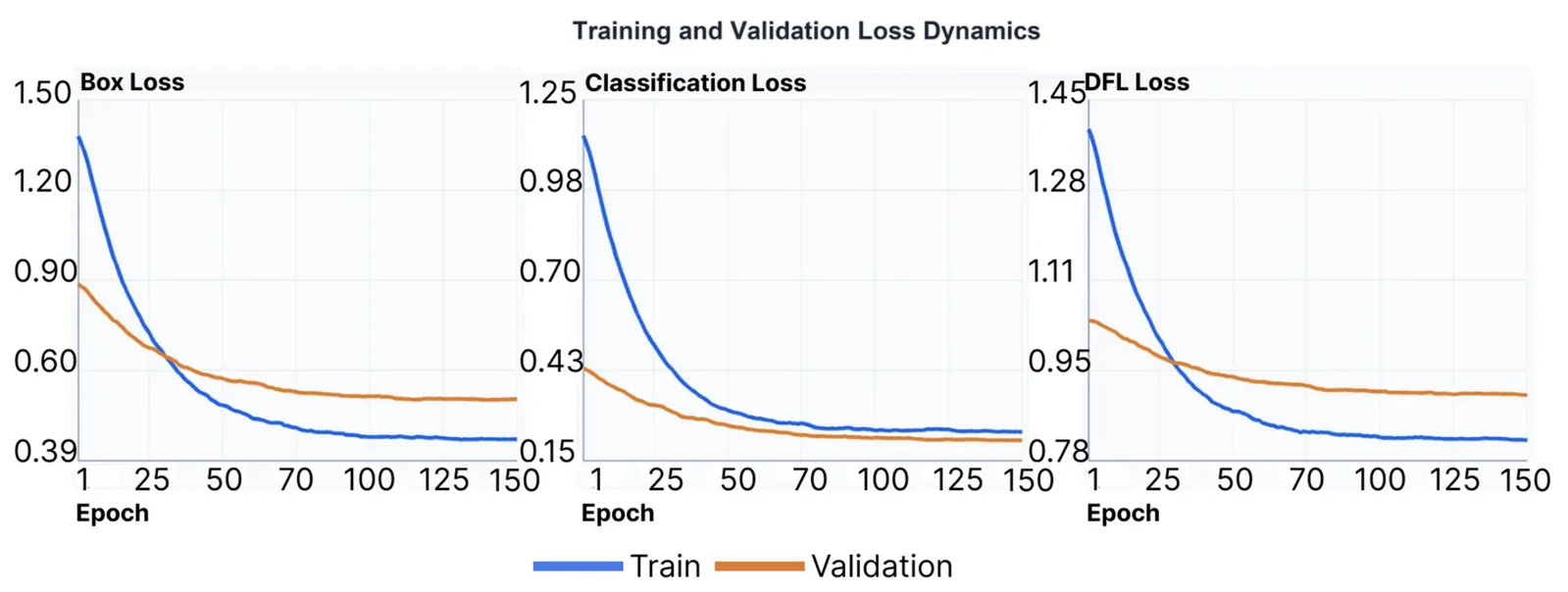
Dynamics of the model’s loss functions on training and validation samples over 150 epochs: Box Loss Localization Error, Classification Loss, and Distribution Focal Loss (DFL Loss).

Figure 7 shows a number of graphs reflecting the values of the loss functions: box_loss characterizes the error in determining the coordinates of the bounding boxes, cls_loss characterizes the error in classifying objects, and dfl_loss takes into account the probability distribution in locating objects. The abscissa axis in the graphs shows the epoch number of the neural network training. Within each epoch, the values of the quality indicators and the loss function are calculated and recorded once, resulting in one initial value of the indicators for each epoch. The graphs simultaneously show two curves: train, which reflects the actual values of the indicators during training, and validation, which represents the operation of the model on the validation dataset.

As can be seen from the presented graphs, the values of all loss functions decrease significantly in the initial epochs of learning and subsequently stabilize at a low level. This indicates that the model is effectively trained and demonstrates resistance to overfitting.

**Figure 8:**
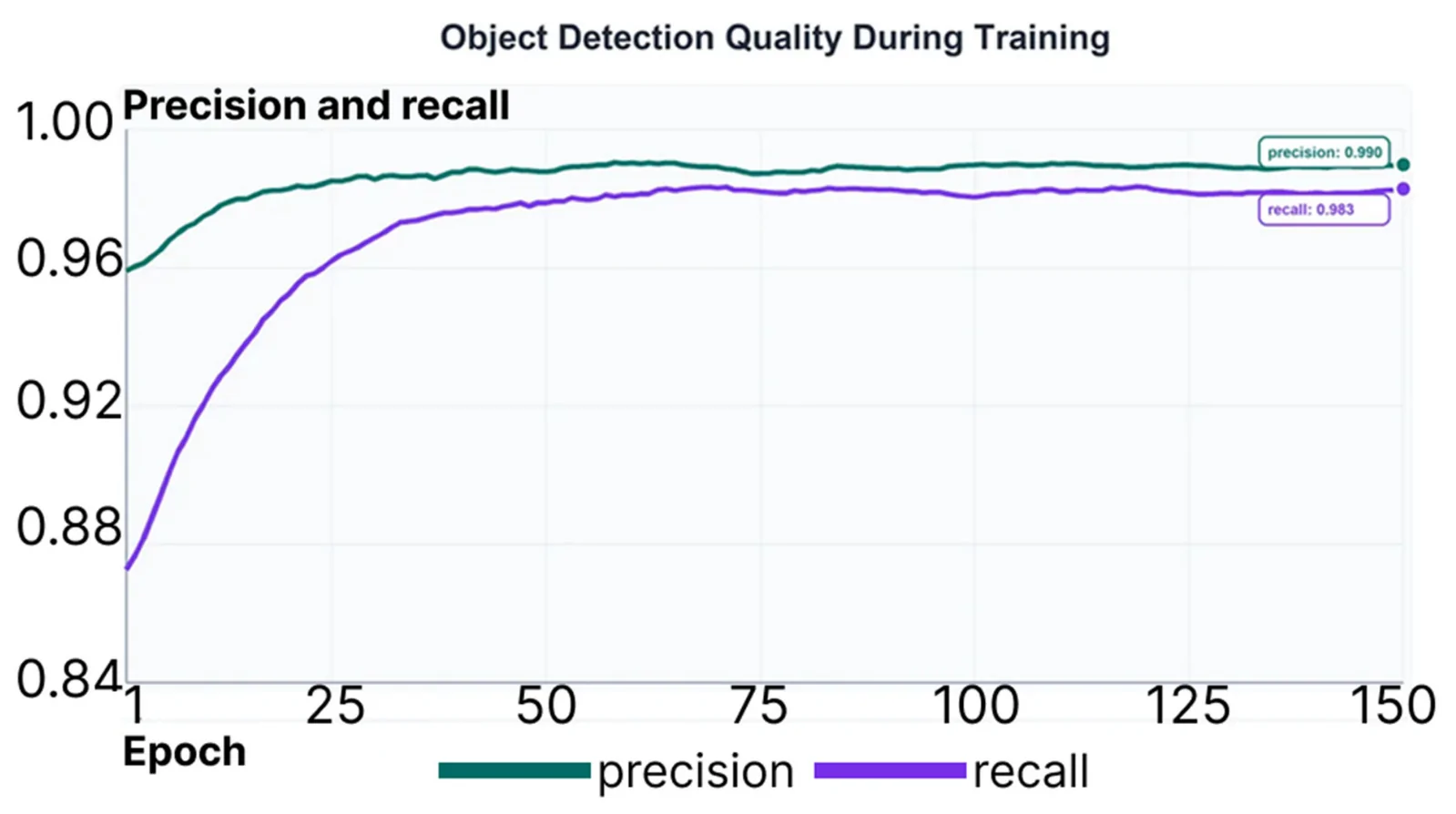
Dynamics of accuracy (precision) and completeness (recall) of object detection in the validation sample over 150 training epochs.

Figure 8 shows the quality metrics of the model—precision and recall. Already at the early stages of training, these indicators reach high values and later demonstrate gradual growth and stability in the range of 0.98–0.99, which indicates the high efficiency of the model and its ability to generalize information when working with real data.

**Figure 9:**
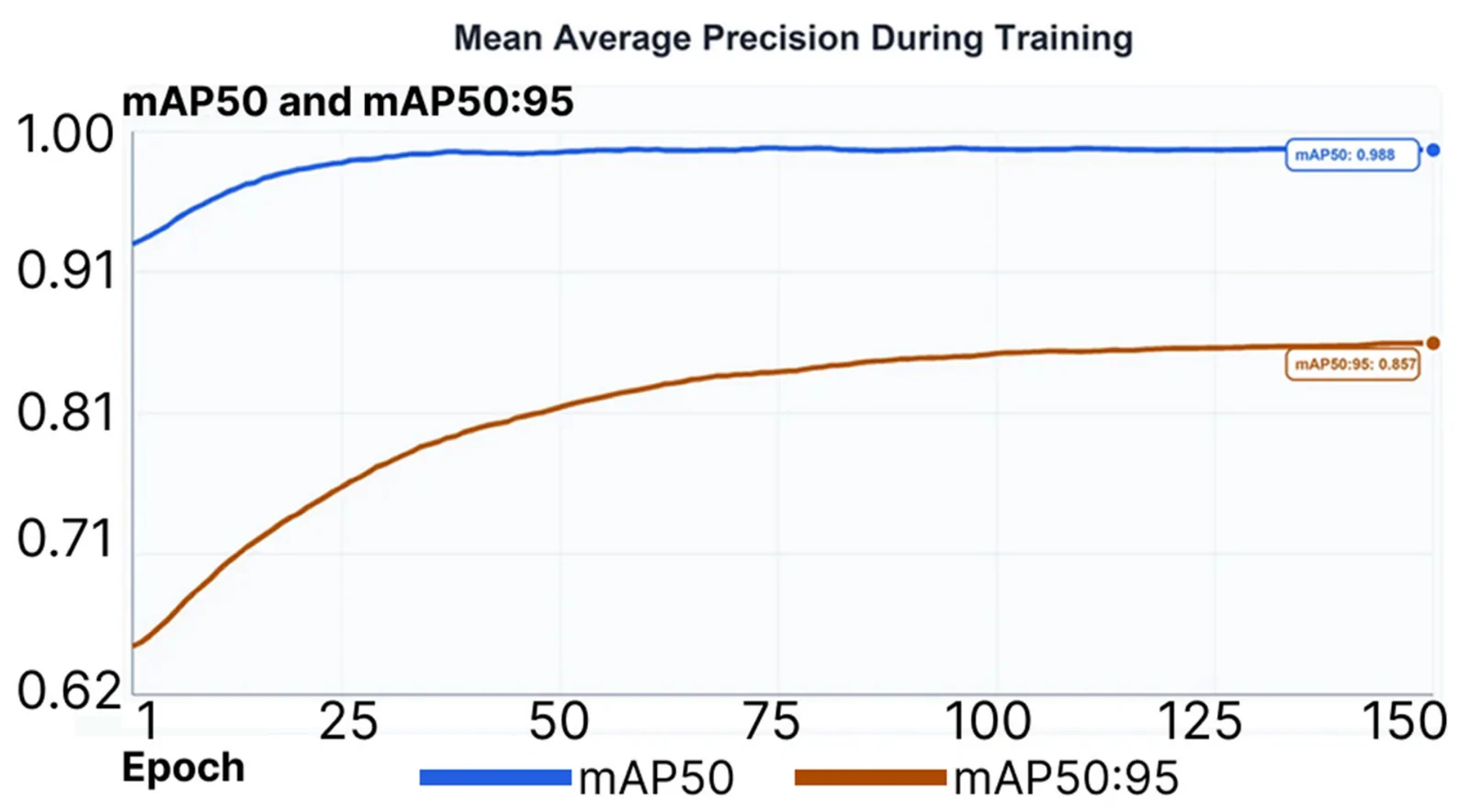
Dynamics of the average accuracy of object detection in the validation sample over 150 epochs of training using the *mAP50* and *mAP50:95* metrics.

Next, Figure 9 shows the main metrics of the *mAP50* and *mAP50:95* computer vision system.

Both metrics show rapid growth in the learning process and reach high values: *mAP50* stabilizes at around 0.98, and *mAP50:95* exceeds 0.85. Such results indicate the high accuracy of the model and are considered very successful in the context of industrial computer vision tasks.

For a more complete analysis of the computer vision model, an experiment was conducted to evaluate the quality of object recognition at different times of the day (Table 2). For this purpose, video recording of technological processes was continuously conducted on the production site of the filling station for two working days. The resulting video stream was divided into homogeneous time intervals of one hour with a frequency of two frames per minute and labeled. As a result, a test dataset was generated, for which a pre-trained neural network was consistently applied.

For each time slot, all frames of the corresponding period were automatically run through the model in order to classify and localize target objects. The average accuracy of the *mAP* at the class level was used as the main metric for recognition quality. The processing results for each time interval were averaged over all observation days. Thus, an aggregated accuracy indicator was formed for each hour of the day.

According to the results of the testing, it was revealed that during the night hours from 0:00 to 6:00, the model demonstrates the worst accuracy, especially for small and complex objects such as the AFS operator and the transition bridge. From 7:00 a.m. to 9:00 a.m., the accuracy of the model stabilizes at around 0.80–0.98 and remains high until noticeable darkening. From 20:00 to 23:00, the accuracy naturally decreases to 0:38 due to night lighting.

**Figure 10:**
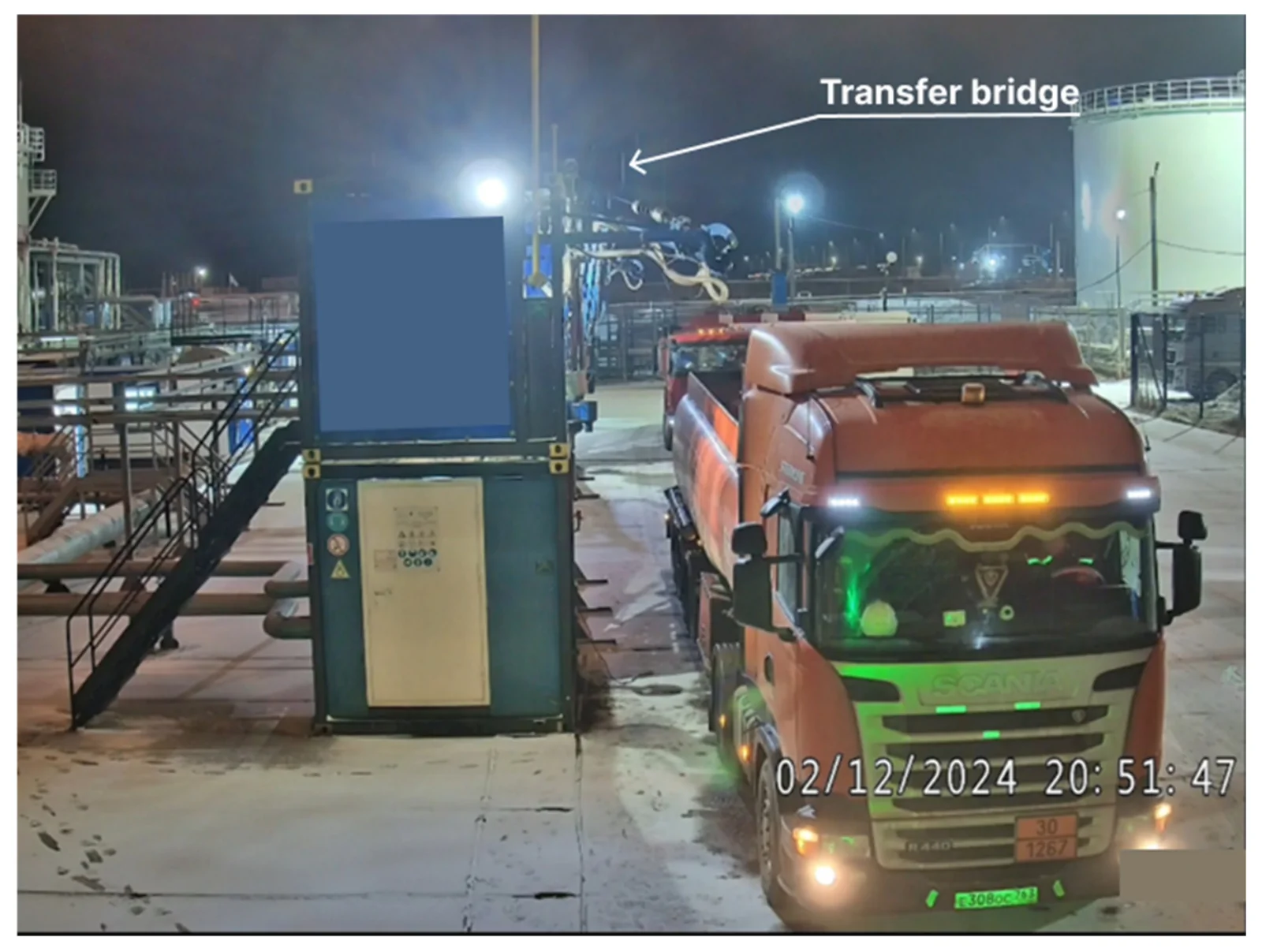
The site of the automated filling system at night.

This decrease in accuracy is not due to the dark time of day, but due to the difficulty of producing night lighting (Figure 10).

The image shows production at night, which is accompanied by pronounced glare in the areas of key facilities. As a result, neither the model recognition algorithm nor the operator is able to reliably identify the desired entities. A similar significant decrease in detection accuracy is observed due to the fact that the model loses the ability to correctly interpret the transition bridge in the garage position, which dominates the other position required for analysis.

At the current stage, the system should be considered the most reliable for daytime operation, as well as for sites where the lighting configuration is configured to minimize direct glare towards the camera.

## 5. Making Decisions About Fixing Events

To determine events at the filling station, it is necessary to interpret the output values of the neural network detection based on an analysis of the position of objects and their condition over time.

**Figure 11:**
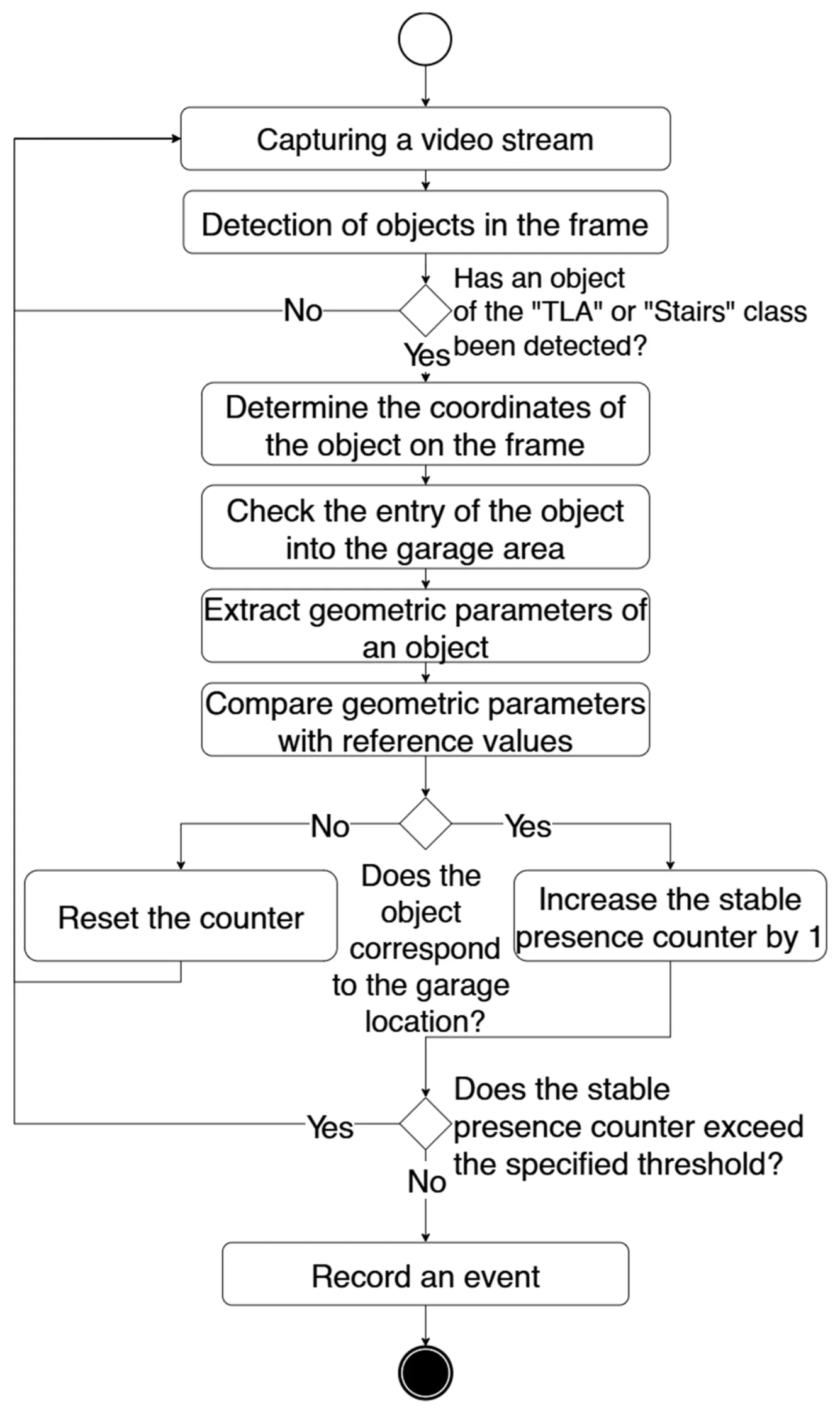
The algorithm for determining the garage position for objects of the class “TLA” and “Transfer bridge”.

The position of a “TLA” class object in the “garage” state is recorded by the system by analyzing the video stream using object detection algorithms (Figure 11). To identify this event, an object classified as a “TLA” is searched on the frame and its localization within a predefined area corresponding to the garage area is checked. Additionally, the geometric characteristics of the object are compared with reference parameters preset for the “garage” state.

If an object is stably fixed in the specified zone for a certain time interval and its dimensions correspond to the reference values, the event is considered confirmed. This approach reduces the number of false positives caused by short-term errors in the detection algorithm.

A similar method is used to determine the position of an object of the “transition bridge” class in a garage. In this case, the garage position indicator is the high ratio of the height of the bounding box to its width, which is typical for a vertically oriented bridge. If such a ratio is consistently detected within a given area and for a certain time, the event is considered fixed.

The determination of the tanker’s grounding is implemented by receiving a signal from the AFS station about connection to the tanker. This process does not require the use of computer vision algorithms and is carried out solely based on data from an external system.

**Figure 12:**
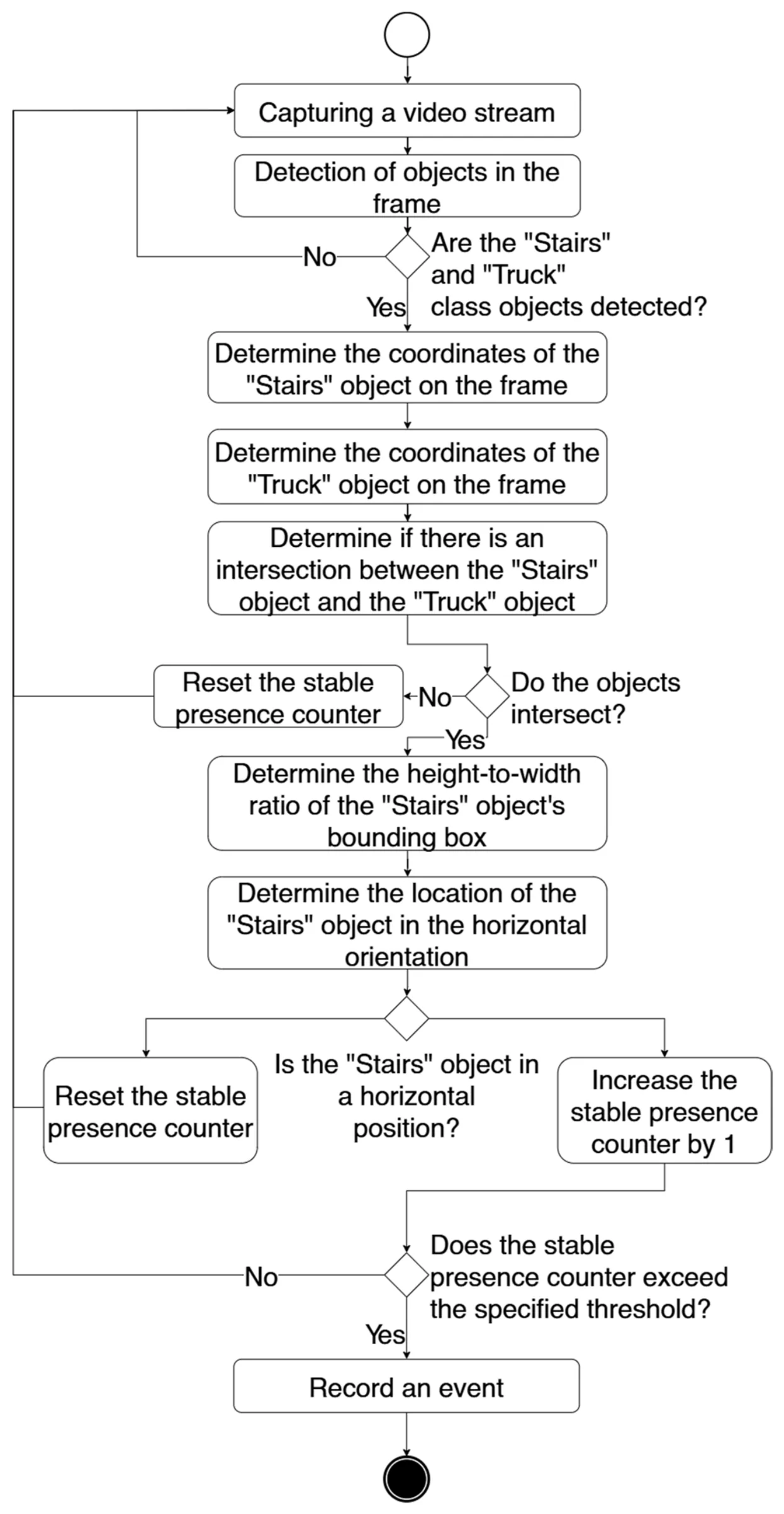
The algorithm for fixing the event “The transfer bridge is installed on a tanker truck”.

To record the event “The transition bridge is installed on a tanker truck”, a method is used to analyze the spatial location of objects detected using computer vision algorithms (Figure 12).

In particular, the intersection of the bounding rectangles of objects classified as “stairs” and “truck” is determined. Additionally, the height-to-width ratio of the bounding box of the transition bridge is checked in order to confirm its horizontal position, which is characteristic of the state of installation on a tanker truck. While meeting the intersection conditions and observing the geometric parameters, the system registers the event “The transition bridge is installed on a tanker truck”.

**Figure 13:**
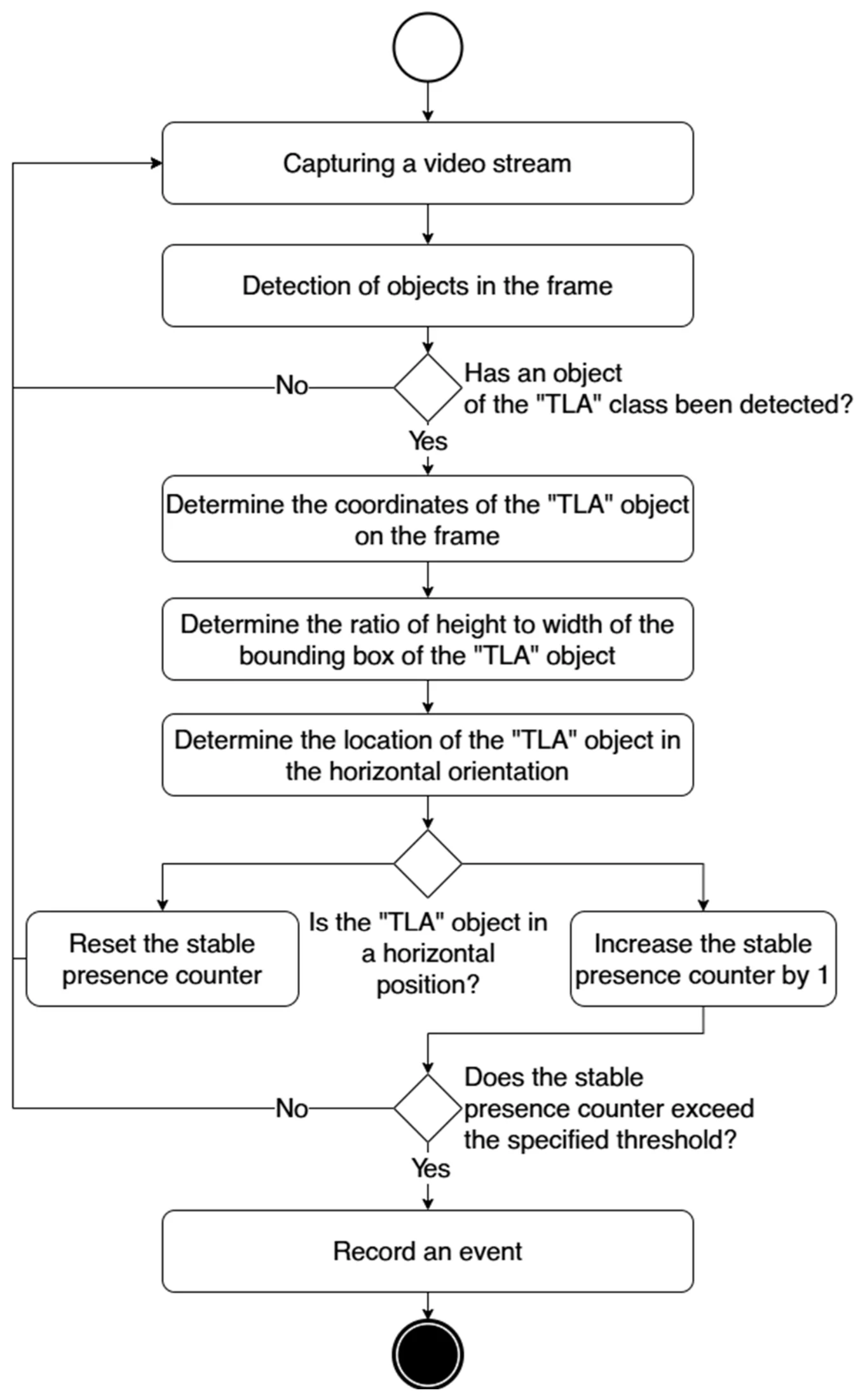
The algorithm for fixing the “TLA installed in a tanker truck” event.

The next event to be monitored is “TLA installed in tanker truck”. To determine it, the geometric parameters of the bounding box of an object classified as a “TLA” are analyzed (Figure 13).

If the ratio of length and width corresponds to the parameters characteristic of the horizontal position, and if this position is maintained steadily for a given number of frames, the system interprets the situation as the occurrence of the specified event.

The “TLA in the hands of the operator” event is considered to have occurred if the following conditions are met for a specified number of consecutive frames: the statements “TLA installed in a tanker truck” and “TLA in a garage position” simultaneously assume a false value. Thus, the fact that the operator holds the TLA is determined by exclusionary logic in the absence of its binding to fixed states.

The regulatory events that determine the beginning and end of the production cycle are “Tanker truck on AFS” and “Tanker truck left AFS”, respectively. To detect them, it is enough to track the presence or absence of an object of the “Truck” class on the frames using computer vision algorithms. Similar to other events, fixation is performed only with steady confirmation: the system must detect or not detect the object for a set number of consecutive frames. This makes it possible to offset the effect of time lapses caused by detection errors or partial overlap of objects in the frame.

All events in the system are represented as the final states of objects. Information about the current and previous states is stored for each monitored object. When a state changes, the system verifies its stability over time: the new state must be maintained for at least five consecutive frames before it is confirmed. For events related to the interaction between objects, an additional criterion is used: the IoU (Intersection over Union) of the bounding boxes must be greater than or equal to 0.2. Additionally, a two-state hysteresis rule is applied to prevent rapid switching between neighboring states.

The rule-based event interpretation algorithm was further tested on 186 complete sequences of operations manually annotated by domain experts. These validation samples were independent of the test samples used to evaluate object detection at the image level. For the complete sequences, the algorithm achieved an accuracy of 0.92 events, completeness of 0.89, and an F1 measure of 0.90. Most errors were related to partial closure of operators or simultaneous closure of overhead filling devices and transition bridges.

**Figure 14:**
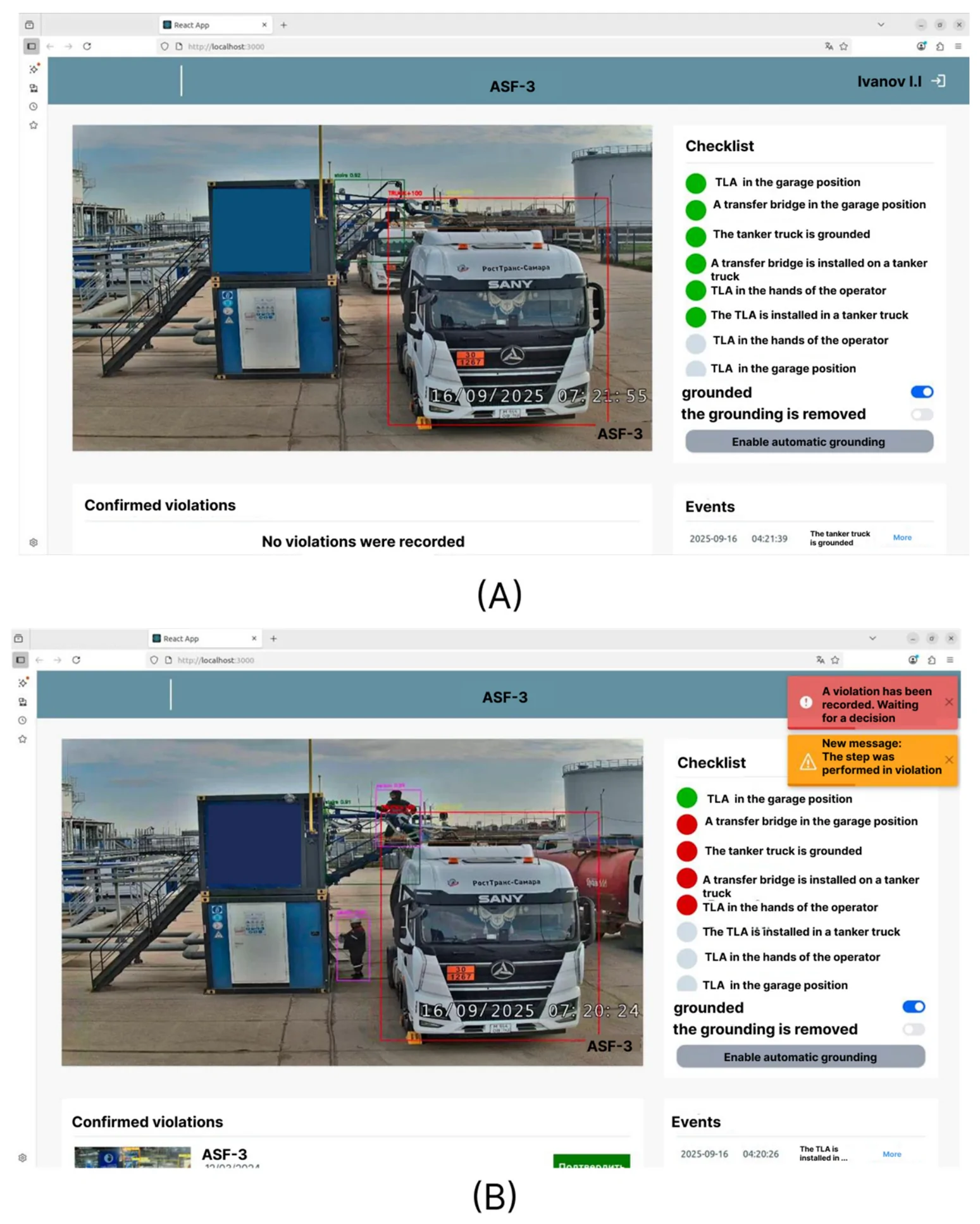
Interface of the software control system for manual operations: (**A**) interface without sequence violations; (**B**) an interface with sequence violations. The green box indicates the object: Stairs, the red box: Truck, the purple box: Person, the yellow box: TLA.

As a result, the developed system ensures the identification of key production events on the site, based solely on sustained and statistically significant environmental changes. This approach allows us to consider the system as a basis for subsequent automation, operational monitoring and analysis of technological processes. Figure 14 shows an example of the functioning of a system with a user interface that clearly confirms the correctness or error of the detected sequence of events.

The final system provides automatic fixation of the stages of the technological process and generates notifications in case of violation of their sequence. Additionally, video footage is recorded of each detected deviation from the established chronology of events for subsequent analysis. The software automates the monitoring of compliance with regulated operations and helps to increase the level of safety when performing oil filling operations.

## 6. Discussion and Analysis of the Results

As a result of the development efforts, we have created a software system that provides the ability to monitor and record the sequence of manual operations performed by employees in a technological process. This solution is focused on ensuring strict control over compliance with production regulations.

The system’s functionality allows the process to be monitored in real-time, which helps us to detect deviations from standards promptly. This contributes to a reduction in the likelihood of violations of technological discipline during operations involving petroleum products.

The final model was tested using recordings obtained in a production environment during different times of day. Experiments revealed a significant decrease in the accuracy of the neural network at night, due to artificial lighting, glare, and reflections in the camera’s field of view. At this stage, the system is most reliable for daytime use, as well as in locations where the lighting setup minimizes direct glare on the camera.

For a full-fledged, round-the-clock implementation, several technical measures are planned to be considered: pre-processing of images in low light conditions, histogram or contrast-limited adaptive histogram equalization (CLAHE), glare suppression filters, changes in the location of cameras and lights, and polarizing filters. In cases where RGB cameras are not sufficient, the use of near-infrared or thermal imaging will also be considered. Prior to the experimental verification of these measures, nighttime monitoring should be treated as an operational limitation of the current prototype.

The 40 h operation of the prototype on a real production site was conducted as a separate phase of field testing and was not included in the sample of images used for testing. During this time, 74 notifications of deviations from approved regulations were recorded; subsequent manual verification confirmed 67 of these as actual violations and 7 as false alarms. The control log, which includes 76 manually identified process deviations, revealed 67 events detected by the system, corresponding to an accuracy of 91%, completeness of 88%, and an average false alarm rate of 1.8 notifications per hour.

In comparison with the existing approaches used in the field of industrial safety, the system proposed in this paper is characterized by a fundamentally new conceptual framework. In most of the known solutions [2,3,4,5,6,7], researchers’ attention is mainly focused on the task of detecting the presence or absence of certain objects in the frame, which, although it provides a basic level of control automation, significantly limits the functionality of such systems. Such solutions, as a rule, are focused on the static interpretation of visual data and do not take into account the temporal dynamics of events, which reduces their effectiveness in analyzing complex production scenarios.

In contrast, the proposed technique is aimed not only at identifying objects, but also at tracking the sequence of human actions in real time. This approach involves the construction of a context-dependent model of operator behavior, which makes it possible to record not only the presence of process elements in the frame, but also the nature of their interaction in dynamics. The implementation of such a mechanism provides a higher level of analytical detail, expanding the system’s capabilities in terms of monitoring compliance with technological regulations and identifying potentially dangerous deviations from regulatory procedures.

In the further stages of the project development, it is planned to expand the functionality of the developed system by increasing the number of controlled objects and interaction scenarios. In particular, it is planned to expand the list of detectable and analyzed elements of the technological process, which will ensure a more complete coverage of the production environment and increase the accuracy of interpretation for operator actions. This is necessary to more accurately monitor the progress of the process and increase the scenarios of deviations from the regulations.

One of the key areas of further development is the introduction of multi-camera monitoring, which ensures monitoring of employees’ actions from several angles simultaneously. This will allow us to form a more complete picture of the spatial context of the events taking place and increase the reliability of the analysis, especially in conditions of partial overlap of facilities or a complex configuration of the production space.

In addition, automated monitoring of multiple filling stations operating simultaneously is considered a potential future expansion of the system. While the current validation was conducted at a single station, multi-site monitoring should be seen as a scalable direction rather than a confirmed outcome of this study. Implementing the system at multiple locations would require a different architecture for camera synchronization, event recording, network stability, and centralized notification to operators.

## 7. Conclusions

The work identified the need to ensure control over compliance with safety requirements at production facilities. Special attention is paid to modern approaches to the implementation of security control using computer vision tools. It has been established that the main part of the existing solutions is mainly focused on monitoring the use of personal protective equipment (PPE) and emergency notification, while the issue of monitoring the correctness of the sequence of technological operations remains insufficiently developed.

Within the framework of the chosen subject area, the procedure for filling petroleum products at an oil refinery was modeled using BPMN notation. For a comprehensive description of the technological process under consideration, a generalized contextual model was developed that reflects the key stages of the filling operation. The specified model was decomposed according to the main phases of the process into four separate business process diagrams.

Based on the developed description of the production process within the chosen subject area and based on the constructed process and contextual models, the identification of key events of interest from the point of view of ensuring compliance with regulatory requirements was carried out.

In order to detect and classify objects involved in controlled events, a neural network model of the YOLO family was trained, characterized by a high processing speed and high accuracy of object localization. To train the model, we used a specialized dataset containing more than 6000 images, labeled into four different classes of objects of interest.

At the next stage of development, based on the output data of the trained computer vision model, a system of interpretation and comparison of detected objects with events described in the business models of the process was implemented. This approach made it possible to link visually recognizable objects with the corresponding stages of a production operation.

The final system demonstrated stable performance in daytime operation, thereby confirming compliance with the declared functional characteristics. Currently, it is being actively validated in the production environment, which makes it possible to assess the reliability and adaptability of the software to the real conditions of the technological process. An indirect but significant indicator of the effectiveness of the system is the number of detected violations of the regulations. This parameter can be considered an objective criterion for the effectiveness of a software product, since the recorded deviations reflect an actual increase in the level of control and detection of potentially dangerous actions by employees.

In the future, it is planned to refine computer vision algorithms in order to improve the accuracy of the system’s operation in low-light conditions. The planned improvements will be implemented by expanding the training data sample to include images recorded at different time periods of the day and at different light levels.

Additionally, the possibility of adaptive adjustment of algorithms for interpreting output parameters is being considered, depending on the actual degree of illumination of the production site. This approach will increase the system’s resistance to external factors and ensure the correct detection of personnel actions at night.

## Figures and Tables

**Table 2:** Estimation of the accuracy of the computer vision model depending on the time of day.

Time of Day	*mAP* for Object Recognition			
	«TLA»	«Truck»	«Person»	«Transfer Bridge»
00:00:00	0.53	0.70	0.42	0.33
01:00:00	0.54	0.67	0.25	0.00
02:00:00	0.48	0.60	0.25	0.00
03:00:00	0.50	0.60	0.25	0.00
04:00:00	0.55	0.70	0.42	0.00
05:00:00	0.61	0.86	0.80	0.00
06:00:00	0.76	0.93	0.92	0.31
07:00:00	0.75	0.97	0.93	0.58
08:00:00	0.86	0.98	0.93	0.69
09:00:00	0.86	0.98	0.92	0.93
10:00:00	0.88	0.97	0.94	0.91
11:00:00	0.85	0.98	0.94	0.97
12:00:00	0.87	0.95	0.94	0.98
13:00:00	0.86	0.97	0.94	0.94
14:00:00	0.89	0.97	0.94	0.95
15:00:00	0.87	0.98	0.93	0.96
16:00:00	0.87	0.98	0.92	0.98
17:00:00	0.88	0.96	0.92	0.98
18:00:00	0.86	0.98	0.89	0.93
19:00:00	0.80	0.96	0.84	0.95
20:00:00	0.81	0.97	0.78	0.89
21:00:00	0.75	0.96	0.78	0.88
22:00:00	0.61	0.87	0.53	0.54
23:00:00	0.58	0.75	0.42	0.40

## Data Availability

The original contributions presented in this study are included in the article. Further inquiries can be directed to the corresponding author.

## Abbreviations

The following abbreviations are used in this manuscript:

PPE	Personal protective equipment
BPMN	Business process model and notation
AFS	Automated filling system
TLA	Top loading arm
IoU	Intersection over union
